# Supplementary material for: Citizens’ perspectives on relocating healthcare
Source: PLoS One. 2024 Aug 29;19(8):e0309382. doi: 10.1371/journal.pone.0309382 (PMC11361562; doi:10.1371/journal.pone.0309382)
Supplement: S1 File — (DOCX) [file pone.0309382.s001.docx]

**Appendix A - Questionnaire**

| **K. The right care in the right place** |
| --- |

To maintain the high quality and accessibility of care in the Netherlands, care must in future be organised differently. Care professionals and the government therefore want to organise **the right care in the right place**.

45 What do you think about when you think about the right care in the right place?

…………………………………………………………

46 What do you think is an example of the right care in the right place? You may give several examples.

…………………………………………………………

47 What do you think is an example where the right care is not in the right place? You may give several examples.

…………………………………………………………

**Appendix B – Time schedule citizen platform**

| Time schedule citizen platform 1 - 25-03-2022 | | | |
| --- | --- | --- | --- |
| **Start time** | **End time** | **Subject** | **Aim/method** |
| 9:00 | 9:30 | Start |  |
| 9:30 | 9:40 | Introduction | Introduction, explanation of the programme and schedule of the day |
| 9:40 | 10:00 | Introduce | Getting to know each other. Creating a safe and open atmosphere. |
| 10:00 | 10:30 | Important aspects in care delivery. | Examine what citizens consider important in care delivery. Participants receive five cards with aspects. They can put these in order of importance. A sixth card is empty on which they can write their own aspect. Next, the participants are asked why this order has been chosen and why an aspect is considered important, or more important. |
| 10:30 | 10:45 | Associations with *the Right Care in the Right Place* | To examine what the participants think of when they think of *the Right Care in the Right Place* and being involved in the right care in the right place, by making a word cloud based on the answers the participants provide via mentimeter. |
| 10:45 | 11:00 | Making urgency clear | Inform participants of the challenges facing the healthcare system and the urgency of the issue through a presentation by Nivel. |
| 11:00 | 11:15 | Break |  |
| 11:15 | 11:25 | Examine if urgency is clear | Check whether the urgency of the problem is clear to the participants, and ask them what solution they have in mind, and what conditions must be met in order for that solution to be realised. The answers are written on a sticky note and stuck on a large sheet of paper. The answers are collected and discussed. |
| 11:25 | 11:35 | Presentation by the Ministry of Health, Welfare and Sport | The Ministry gave a presentation about the movement *the Right Care in the Right Place*. |
| 11:35 | 12:05 | Expectations and assumptions | For another, related, study on *the Right Care in the Right Place*, a group discussion was used to examine what the participants expect from being involved in on *the Right Care in the Right Place*. |
| 12:05 | 12:25 | The relocation of secondary care to primary care | To examine what citizens think about relocating care from the hospital to the GP and what conditions must be met. Cases are presented and the group splits up into three smaller groups who then discuss the cases. |
| 12:25 | 13:10 | Lunchbreak |  |
| 13:10 | 13:30 | Relocating care from the GP to other providers | To examine what citizens think about relocating care from the GP to other providers and what conditions must be met. Two cases are presented and participants write down their answers on a sticky note. Participants were asked what they would do if they were the person in the case. In the discussion, they tell why they would choose a certain way of acting. |
| 13:30 | 13:50 | Relocating care from the GP to self-care | Examining what citizens think about relocating care from the GP to self-care. Four cases are presented and the participants indicate whether they would go to the GP or not and why. There will also be a discussion on what options there would be other than going to the GP and how to increase self-care. |
| 13:50 | 14:10 | The role of citizens | For another, related, study on the right care in *the Right Care in the Right Place*, we seek to find out what roles citizens see for themselves in *the Right Care in the Right Place*. Four possible roles are presented and participants each fill in a matrix. |
| 14:10 | 14:25 | Break |  |
| 14:25 | 14:55 | Ways and situations of being involved | A discussion of the completed matrices. Discussing what it takes to participate more and when the need is felt to be involved. |
| 14:55 | 15:20 | Approach | For another, related, study on *the Right Care in the Right Place* we seek to find out how participants want to be approached, and by whom, about *the Right Care in the Right Place*. Four groups are formed. Each group is given a role for the citizen and the questions are discussed. |
| 15:20 | 15:30 | End | Evaluation |

| Time schedule citizen platform 2 01-04-2022 (online) | | | |
| --- | --- | --- | --- |
| **Start time** | **End time** | **Subject** | **Aim/method** |
| 9:00 | 9:30 | Connect with zoom |  |
| 9:30 | 9:40 | Introduction | Introduction, explanation of programme, and schedule of the day |
| 9:40 | 9:55 | Introduce | Getting to know each other. Create a safe and open atmosphere. |
| 9:55 | 10:10 | Important aspects in care delivery | We examine what citizens consider important in care delivery. Participants fill in a Google form in which they can rank five aspects of care delivery. They can add the sixth aspect of their choice. Participants are then asked, why this order has been chosen and why an aspect is considered important, or more important. |
| 10:10 | 10:25 | Associations with *the Right Care in the Right Place* | To examine what the participants think of when they think of *the Right Care in the Right Place* and being involved in *the Right Care in the Right Place*, by making a word cloud based on the answers the participants provide via Mentimeter. |
| 10:25 | 10:40 | Making urgency clear | Inform participants of the challenges facing the health care system and the urgency of the issue through a presentation by Nivel |
| 10:40 | 11:00 | Present and discuss a possible solution through relocating care. | Examine how citizens think about relocating care. We asked what care could be relocated and what conditions must be met to relocate care? Participants can write down their answers in Mentimeter. Once everybody has answered the answers are discussed.  The above questions are asked both for relocating care from the hospital to the GP as well as relocating care from the GP to self-care. |
| 11:00 | 11:15 | Break |  |
| 11:15 | 11:30 | Presentation by the Ministry of Health, Welfare and Sport | To clarify the position and policy of the ministry regarding the right care in the right place. Indicating what will be done with the results of the citizen platform. |
| 11:30 | 11:45 | Role citizens | For another, related, study on *the Right Care in the Right Place*, we seek to find out what roles citizens see for themselves in *the Right Care in the Right Place*. Four possible roles are presented and participants each fill in an online matrix. |
| 11:45 | 12:05 | The ways and situations of being involved | A discussion of the completed matrices. Discussing what it takes to participate more and when the need is felt to be involved. |
| 12:05 | 12:25 | Approach | For another, related study on *the Right Care in the Right Place* we seek to find out how participants want to be approached, and by whom, about *the Right Care in the Right Place*. Four groups are formed. Each group is given a role for the citizen and the questions are discussed. |
| 12:25 | 12:35 | End | Evaluation |

**Appendix C - Detailed information programme for the citizen platforms**

The first step during the citizen platforms was to examine what participants found the most important aspect of care delivery. Participants during the first citizen platform had six cards. Five contained an aspect, concerning the following five aspects: familiarity with the caregiver, expertise, distance, costs for the patient, and waiting time. The aspects were derived from studies on patient satisfaction with regard to the location of care [14-16, 18]. The sixth card was empty. On these, participants were allowed to write down an aspect themselves. Participants were asked to place the cards in order from most important to the least important (range 1 through to 6). Participants during the second citizen platform did not have physical cards but could place an order online. Secondly, the participants were asked what they considered to be *the Right Care in the Right Place*. They could write this down in Mentimeter.

Thirdly, a presentation was given by Nivel. The presentation was about the developments taking place in society and the problems this poses for the sustainability of the healthcare system. After this

the participants were asked what solutions they had in mind to solve the problem of the unsustainable healthcare system. This exercise was been carried out during the first citizen platform.

During the first citizen platform, there were assignments about relocating care whereby the participants were presented with cases. All cases have been submitted to a GP for review and she had also contributed to the cases and devised a number of cases. It was decided to use cases to make the subject less abstract. Additional questions about relocating healthcare in general were asked after presenting the cases.

The first topic discussed was relocating care from the hospital to the GP. Participants were divided into three groups and each group was presented with a different case. The cases concerned the removal of stiches, follow-up cancer examinations, and an apnoea examination. Participants were asked whether they would go to the GP or to the hospital (See Box 1 for the cases).

**Box 1 – Cases about relocating care from the hospital to the GP***Case 1*You underwent a knee prosthesis two weeks ago. It was a planned surgery because of the wear to your knee. The operation went well and, during your recovery at home, there were no unexpected complications. The stitches in your knee could be removed after two weeks. You can have the stitches removed by your general practitioner or at the hospital. Where would you have the stitches removed?

*Case 2*
You were diagnosed with bowel cancer three years ago. It was possible to remove the tumour and no metastases were found. To make sure that the cancer does not return, you must have blood tests twice a year and an ultrasound scan of your abdomen once a year. The blood and ultrasound tests are carried out in the hospital. You can get the results of the tests from your general practitioner: they will discuss the results with you and refer you to the hospital if any abnormalities are found. You can also get the results from the hospital. With whom would you like to discuss the results of the tests?

*Case 3*Your partner complains of disturbed sleep because you snore. You, yourself, also have complaints about it. You are tired during the day and suffer with poor concentration. Your general practitioner advises you to have an examination to check for sleep apnoea, causing pauses in breathing during sleep, which, apart from snoring, can also cause complaints during waking hours. The general practitioner tells you that you can choose to have the examination in the hospital, but that it can also be carried out at home nowadays. If you choose to have the examination in the hospital, you will stay there overnight. The hospital staff will apply sensors to your body that are necessary for the examination. You will receive the results of the examination from a specialist at a later date. If you choose to have the examination at home, the necessary equipment and instructions will be sent to you by post. You must attach the sensors to your body yourself. The instructions clearly explain how to do this. A specialist will assess the results and discuss them with you. Do you choose to have the test at home or at the hospital?

The next topic discussed was the shift of care from the GP to other primary care professionals. Cases about an individual with back pain and an individual with obesity were given, saying that the individuals in the case would like to have professional help. The question to the participants was: how would you approach this? See box 2 for the cases.

**Box 2 – Cases about relocating care from the GP to other HCPs***Case 1*You have been suffering with lower back pain for a few weeks. The pain is increasing and, as a result, you are becoming increasingly immobile. Because the complaints do not seem to disappear on their own, you would like a professional to look at it. How would you approach this?

*Case 2*You are overweight. You struggle with this and you do not feel comfortable with it. You would like to do something about your weight and you are searching for professional help. How would you approach this?

The last topic discussed was about relocating care from the GP to sustainable self-care. Four cases were presented concerning a child with a fever, a man with diarrhoea, a women with painful ankles and a women with loneliness. Participants were asked if they would go to the general practitioner or not and why (See Box 3).

**Box 3 – Cases about relocating care from the GP to self-care***Case 1*You have a one-year-old child. For two days now your child has had a 40 degree temperature, a cough, and a running nose, though no tightness in breathing. Otherwise, your child is behaving as usual. Your child does not have Covid-19. What would you do?

*Case 2*You are a healthy thirty-year-old man with diarrhoea. It started yesterday mid-morning and soon you needed to use the toilet every hour for most of the day. Your stomach is very upset, but you do not vomit. You drink well but have little appetite. At work, some of your colleagues have similar symptoms. Today, at lunchtime, you have had diarrhoea a few times. What would you do?

*Case 3*You are a 55-year-old woman and you returned from a winter sports holiday yesterday. You skied for the first time after 15 years. Your ankles hurt a lot. What would you do?

*Case 4*You are a 40-year-old woman and you feel lonely. Your feelings of loneliness are getting stronger and will not go away. You are not gloomy or depressed. You would like help but are also embarrassed to talk about your feelings of loneliness. What would you do?

After the first citizen platform, it was decided to obtain some more information about relocating healthcare from the hospital to the GP and from the GP to self-care. This was achieved by asking which care could be relocated and under which circumstances. Participants could type their answers into Mentimeter and thereafter there was a discussion about the answers they gave.

**Appendix D – Themes identified in the questionnaire**

1. Quality of healthcare/specialized healthcare

- Specialised HCPs/high-quality healthcare
- Centralisation of specialisms in hospitals
- Decentralisation of specialisms across multiple hospitals
- Preference for high-quality care over proximity to home

2. Costs

- Affordable care

3. Accessibility

- Care close to home
- Eliminating waiting lists and ensuring availability

4. Right care provider

- The right HCP for the right question
- GPs can take over tasks from hospitals
- GPs should not take over tasks from the hospital

5. No unnecessary care

- Apply self-care when feasible
- Avoiding overuse the GP’s out of hours service

6. Patient-centred care

- Listen to the patient
- Shared decision making

7. Collaboration between HCPs

- Good communication between HCPs
